# Simultaneous absolute protein quantification of seven cytochrome P450 isoforms in rat liver microsomes by LC-MS/MS-based isotope internal standard method

**DOI:** 10.3389/fphar.2022.906027

**Published:** 2022-08-17

**Authors:** Fulin Jiang, Chang Zhang, Zihan Lu, Jingyu Liu, Peiqing Liu, Min Huang, Guoping Zhong

**Affiliations:** ^1^ Institute of Clinical Pharmacology, Guangdong Provincial Key Laboratory of New Drug Design and Evaluation, School of Pharmaceutical Sciences, Sun Yat-sen University, Guangzhou, China; ^2^ School of Pharmaceutical Sciences, National and Local United Engineering Lab of Druggability and New Drugs Evaluation, Sun Yat-sen University, Guangzhou, China

**Keywords:** cytochrome P450, absolute quantification, LC-MS/MS, rat liver microsomes, isotope internal standard

## Abstract

The cytochrome P450 (CYP) enzymes play a pivotal role in drug metabolism. LC-MS/MS-based targeting technology has been applied to the analysis of CYP enzymes, promoting drug development and drug-drug interaction studies. Rat is one of the most commonly used models for drug metabolism assessment, but LC-MS/MS assay quantifying the abundance of CYP enzymes in rats is rarely reported. Herein, an accurate and stable LC-MS/MS based method was developed and validated for the simultaneous quantification of seven major rat CYP isoforms (CYP1A2, 2B1, 2C6, 2C11, 2D1, 2E1, and 3A1) in liver microsomes. The careful optimization of trypsin digestion and chromatography combined with isotope-labeled peptide as internal standard improved the efficiency and accuracy of the analysis. Highly specific surrogate peptides were obtained by a procedure including trypsin digestion for six hours and separated on a Hypersil Gold C18 column (100 × 2.1 mm, 3 μm) using gradient elution for 15 min with a mobile phase of water containing 0.1% formic acid and acetonitrile. In the method validation, linearity, matrix effect, recovery, stability, accuracy, and precision all meet the requirements. Subsequently, this method was applied to detect seven enzymes in rat liver microsomes from four different sources, and the correlation between the abundance and activity of CYP enzymes was further analyzed. The high-throughput detection method provided in this study will provide support for pertinent pharmaceutical research based on rat models.

## 1 Introduction

The cytochrome P450 (CYP) superfamily is an important enzyme system that mediates a major proportion of phase I metabolism of most drugs, xenobiotics, and endogenous compounds. CYPs are expressed in various tissues and organs, and the liver tissue has the most abundant (Zanger and Schwab, 2013). The expression and activity of drug-metabolizing enzymes in rat liver microsomes have long been the focus of *in vitro* and *in vivo* evaluation of drug disposition and drug-drug interactions (Singh et al., 2011; Manikandan and Nagini, 2018). The CYPs proteins expressed in rat liver mainly include CYP 1–3 families, which not only biotransform a wide variety of drugs, but can also be induced or inhibited by them (Martignoni et al., 2006). Consequently, reliable methods must be considered to quantify these proteins for drug development and evaluation (Xie et al., 2016).

Several biochemical approaches for the determination of CYP isoforms have been developed, mainly including: Western blotting (WB), enzyme linked immunosorbent assay (ELISA), quantification of mRNA levels using real-time polymerase chain reaction (RT-PCR) and determination of metabolic activities by the probe substrate method. Data comparisons between these different techniques show a great deal of variability, which is the most likely cause of the contradictory results (Dostalek et al., 2011; Grangeon et al., 2019; Ren et al., 2020). WB has the characteristics of a semi-quantitative method, and immunological methods such as WB and ELISA also have limitations including weak antibody specificity, poor reproducibility and lack of high-throughput capability (Li and Zhu, 2020). The RT-PCR method is simple, rapid and highly selective. However, the regulation of mRNA at the translational level and the post-translational modification of proteins are affected by many factors, so measured mRNA levels cannot accurately reflect the expression of CYP enzymes (Pearce et al., 2016; Zhang et al., 2016; Couto et al., 2020). The probe substrate method takes into account the effects of genes and environmental factors on enzyme activity, but due to the questionable specificity of the probe-substrate, this method lacks a unified standard when it is widely used.

Targeted proteomics techniques based on liquid chromatography-tandem mass spectrometry (LC-MS/MS) have been a powerful tool for quantifying CYP enzymes. LC-MS/MS combines the high separation ability of liquid chromatography with the high selectivity and sensitivity of mass spectrometry, and also has a high-throughput capability. Several studies have applied this method to detect CYP enzymes in human liver microsomes (Bhatt and Prasad, 2018). However, there are few reports on the detection of CYPs in rat liver, which is one of the most widely used research models. Shao Y et al. established total protein concentration to establish the “Standard Curve Slope” method to assess the gender difference of CYPs in rat liver microsomes (Shao et al., 2017). Hammer H et al. used a targeted mass spectrometry-based immunoassay to directly quantify CYPs and drug transporters (Hammer et al., 2021). The above methods were not fully validated and may have potential variations in quantification (Wegler et al., 2017). Thus, this study aims to develop and validate a highly accurate and stable LC-MS/MS assay for absolute protein quantification of CYP isoforms in rat liver microsomes. We identified seven major CYPs (CYP1A2, 2B1, 2C6, 2C11, 2D1, 2E1, and 3A1) based on the main metabolic enzymes in the human liver and the species differences between human and rat metabolic enzymes (Martignoni et al., 2006; Zhang et al., 2016). All these CYPs were widely used in the study of drug metabolism (Shao et al., 2017; Hammer et al., 2021). Many challenges can affect protein quantification in a bottom-up proteomic workflow, and the pretreatment process including efficient enzymatic digestion is critical to the robustness and sensitivity of the method. Denaturation, reduction, alkylation and digestion of proteins can all affect the acquisition of surrogate peptides. In addition, the complex composition of biological samples may lead to the matrix effect that also affect peptide quantification (An et al., 2019), so chromatography needs to be optimized for efficient separation, which has led many studies to take more than an hour for an analysis (Grangeon et al., 2019; Wenzel et al., 2021). To correct for the matrix effect, an isotope internal standard is usually chosen because it has similar physicochemical properties to the target peptide. Isotope internal standards can also correct for biases introduced by pretreatment and errors in the injection system (De Nicolo et al., 2017). All of these advantages make the isotope internal standard method the first choice for LC-MS/MS based quantification.

To sum up, we developed and validated herein a quantification assay by LC-MS/MS for simultaneously detecting the abundance of seven CYP enzymes in rat liver microsomes. The isotope-labeled peptide corresponding to the surrogate peptide was used as the internal standard to improve the quantitative accuracy. Finally, this method was successfully applied to detect the expression of CYP enzymes in rat liver microsomes, and a correlation analysis was carried out between the measured abundance of CYPs and their activity detected by the probe substrate method.

## 2 Material and methods

### 2.1 Chemicals and reagents

The male Sprague Dawley rat liver microsomes (Protein content, 20 mg/ml) were obtained from IPhase Pharma Services (Beijing, China), Corning Gentest (Corning, American), PrimeTox (Wuhan, China) and Meilunbio (Dalian, China). Recombinant Trypsin (Porcine pancreas) was provided by Yuanye (Shanghai, China). Bupropion and glibenclamide were purchased from National Institutes for Food and Drug Control (Beijing, China). Phenacetin, acetaminophen, tolbutamide, 4-Hydroxy-tolbutamide, dextromethorphan, dextrorphan chlorzoxazone and 6-Hydroxy-chlorzoxazone were purchased from Sigma-Aldrich Inc (United States). Hydroxy-bupropion, phenytoin, 4-Hydroxymephenytoin, testosterone and 6β-Hydroxy-testosterone were purchased from Zzstandard (Shanghai, China). Ammonium bicarbonate (NH_4_HCO_3_), trifluoroacetic acid (TFA), and formic acid (FA) were purchased from Macklin (Shanghai, China). HPLC-MS grade acetonitrile (ACN) and iodoacetamide (IAA) were purchased from Merck (Darmstadt, Germany). Dithiothreitol (DTT) was provided by Thermo Fisher Scientific (Waltham, United States). Dimethyl sulfoxide (DMSO) was provided by MP Biomedicals (France). Acetic Acid (ACE) was from Guangzhou chemical reagent (Guangzhou, China). Deionized water was generated using the Milli-Q Direct 8 water system (Germany). Surrogate peptides and their stable isotope-labeled internal standards (Table 1) were synthesized by Bankpeptid (Hefei, China). All peptide purity was superior to 95.0% and the concentration/net peptide was determined by amino acid analysis. Standards were weighed using a balance of one ten thousandths from METTLER TOLEDO (Switzerland).

**TABLE 1:** Overview of surrogate peptides and their respective ions and mass transitions used for CYP enzyme quantification (^a^isotope-labeled amino acid).

Protein	Peptide	Molecular weight	Precursor	z	Product	Ion/z	CE (V)	Tube lens (V)
CYP1A2	YTSFVPFTIPHSTTR	1754.0	877.7	2+	698.4	b12/2+	30	110
	YTSFVPFTI*PHSTTR	1761.0	587.9	3+	581.9	y10/2+	20	100
	NFNDNFVLFLQK	1498.7	500.5	3+	647.8	y5/1+	13	78
CYP2B1	FSDLVPIGVPHR	1336.6	446.4	3+	409.1	y3/1+	15	75
	FSDL*VPIGVPHR	1343.5	448.6	3+	409.2	y3/1+	13	86
	EALVGQAEDFSGR	1378.5	690.1	2+	966.0	y9/1+	25	98
CYP2C6	EALIDHGEEFAER	1515.6	506.1	3+	602.2	y10/2+	12	80
	EAL*IDHGEEFAER	1522.7	508.4	3+	601.8	y10/2+	20	80
	EHQESLDVTNPR	1424.5	713.2	2+	703.9	y6/1+	18	100
CYP2C11	YIDLVPTNLPHLVTR	1751.2	584.9	3+	574.7	y10/2+	15	80
	YIDL*VPTNLPHLVTR	1758.2	587.0	3+	574.7	y10/2+	17	93
	EALVDLGEEFSGR	1421.5	711.8	2+	1010.0	y9/1+	22	101
CYP2D1	GTTLIINLSSVLK	1358.7	680.1	2+	446.2	y4/1+	14	90
	GTTL*IINLSSVLK	1365.7	683.6	2+	446.2	y4/1+	14	90
	NLTDAFLAEVEK	1349.6	675.6	2+	906.2	y8/1+	15	97
CYP2E1	FINLVPSNLPHEATR	1708.0	570.2	3+	1121.4	y10/1+	18	86
	FINL*VPSNLPHEATR	1715.0	572.6	3+	1121.9	y10/1+	15	90
	FKPEHFLNENGK	1459.6	487.5	3+	592.8	y9/2+	14	84
CYP3A1	QGLLQPTKPIILK	1448.9	483.9	3+	477.9	–^a^	12	84
	QGL*LQPTKPIILK	1455.9	486.2	3+	479.9	–^a^	12	80

a The daughter ion is the ion after amino-terminal cyclization.

### 2.2 Selection of surrogate peptides

Suitable surrogate peptides for absolute quantification of the aforementioned metabolic enzymes were selected by combining in silico and *in vitro* methods (Bhatt and Prasad, 2018). At first, the respective protein sequences were retrieved from the UniProtKB/Swiss-Prot database and underwent an in-silico trypsin digestion (http://web.expasy.org/peptide mass/). According to the mass range of the MS and to ensure protein specificity, peptides with a sequence length of 7–20 amino acids were considered as suitable candidates (Supplementary Table S1). Several criteria were chosen to establish the principles for selecting peptides: 1) surrogate peptides do not contain cysteine, methionine and/or tryptophan amino acids that can cause oxidative instability; 2) non-synonymous genetic polymorphisms with a frequency <1.0% in the population are required; 3) surrogate peptides with high specificity are preferentially selected by NCBI protein raw search; 4) repeated sequences of arginine and lysine should be avoided due to the risk of missed cleavages by trypsin.

### 2.3 Calibration standard and quality control samples

Stock solutions were prepared by dissolving about 1 mg of surrogate peptides or isotope-labeled peptides to obtain 1 mg/ml in ACN: Water: ACE: DMSO (15:80:5:0.5, v/v). All stock solutions were stored at −80°C. DMSO in the mixed solvent was used to reduce the adsorption of peptides to plastic centrifuge tubes (van Midwoud et al., 2007; Li et al., 2022). The calibration standards were prepared by diluting stock solutions in the mixed solvent to generate analytical ranges of 5–1000 nM for CYP1A2, 2C11 and CYP2D1, 0.5–100 nM for CYP2B1 and 3A1, 2–400 nM for CYP2C6 and 2E1, and the quality control (QC) samples were set according to their respective standard curve ranges (Table 2). An internal standard solution containing all stable isotope-labeled internal standards was prepared and their final concentration was 50 nM except for GTTL*IINLSSVLK (CYP2D1) which was 500 nM. The high concentration of the internal standard was used for CYP2D1 to avoid the response reduction of isotope internal standard due to ion suppression caused by the analyte (Tan et al., 2011; Liu et al., 2019).

**TABLE 2:** The intra- and inter-batch precision and accuracy of QC samples for surrogate peptides. The results of three analysis batches were used for inter-batch calculation. RSD, relative standard deviation; RE, relative error.

Protein	Peptide	Conc	Intra-batch (*n* = 6)			Inter-batch		
		(nM)	Mean ± SD (nM)	RSD (%)	RE (%)	Mean ± SD (nM)	RSD (%)	RE (%)
CYP1A2	YTSFVPFTIPHSTTR	5	4.33 ± 0.37	8.5	−13.3	4.94 ± 0.63	12.8	−1.3
		15	12.99 ± 0.03	0.2	−13.4	13.64 ± 1.04	7.6	−9.1
		150	132.93 ± 3.01	2.3	−11.4	140.43 ± 9.90	7.1	−6.4
		750	805.70 ± 43.89	5.4	7.4	810.98 ± 34.89	4.3	8.1
CYP2B1	FSDLVPIGVPHR	0.5	0.50 ± 0.08	15.6	−0.7	0.50 ± 0.07	13.8	−0.3
		1.5	1.34 ± 0.06	4.2	−10.7	1.46 ± 0.15	10.5	−2.8
		15	14.65 ± 0.57	3.9	−2.4	14.08 ± 0.84	6.0	−6.1
		75	77.99 ± 5.02	6.4	4.0	76.22 ± 5.00	6.6	1.6
CYP2C6	EALIDHGEEFAER	2	2.10 ± 0.29	13.9	5.0	2.21 ± 0.20	9.0	10.6
		6	5.63 ± 0.56	10.0	−6.1	5.67 ± 0.58	10.2	1.3
		60	57.10 ± 5.36	9.4	−4.8	56.22 ± 4.02	7.2	−4.1
		300	318.63 ± 11.45	3.6	6.2	307.89 ± 19.85	6.4	1.0
CYP2C11	YIDLVPTNLPHLVTR	5	4.44 ± 0.41	9.3	−11.2	4.53 ± 0.48	10.5	−8.4
		15	13.31 ± 0.68	5.1	−11.2	14.10 ± 1.21	8.6	−3.4
		150	143.94 ± 7.16	5.0	−4.0	143.73 ± 5.74	4.0	−4.2
		750	744.86 ± 59.31	8.0	−0.7	741.59 ± 54.62	7.4	−1.3
CYP2D1	GTTLIINLSSVLK	5	4.84 ± 0.45	9.3	−3.3	4.75 ± 0.58	12.3	−6.1
		15	13.80 ± 0.68	5.0	−8.0	13.72 ± 0.66	4.8	−8.8
		150	139.44 ± 10.51	7.5	−7.0	135.92 ± 7.45	5.5	−10.6
		750	664.79 ± 21.48	3.2	−11.4	674.67 ± 25.84	3.8	−9.3
CYP2E1	FINLVPSNLPHEATR	2	2.17 ± 0.23	10.8	8.5	1.98 ± 0.28	14.4	−4.3
		6	5.53 ± 0.42	7.7	−7.9	5.72 ± 0.43	7.5	−3.3
		60	56.53 ± 3.09	5.5	−5.8	54.89 ± 4.51	8.2	−-9.9
		300	326.91 ± 19.61	6.0	9.0	311.22 ± 23.83	7.7	0.9
CYP3A1	QGLLQPTKPIILK	0.5	0.46 ± 0.06	13.0	−7.6	0.51 ± 0.08	15.0	2.2
		1.5	1.41 ± 0.19	13.4	−6.0	1.45 ± 0.12	8.3	−3.1
		15	14.04 ± 0.58	4.2	−6.4	14.22 ± 0.59	4.1	−5.2
		75	80.03 ± 2.60	3.3	6.7	76.46 ± 5.11	6.7	2.0

### 2.4 LC-MS/MS analysis

LC-MS/MS analyses were conducted on TSQ Quantum Access Max API mass spectrometer (ThermoFisher Scientific, Massachusetts, United States) with an electrospray ionization (H-ESI) interface coupled to an UHPLC system (ThermoFisher Scientific, Massachusetts, U.S.). Tune Plus® software 2.4 was used to control this instrument.

Chromatographic separation was performed using a C18 (100 × 2.1 mm, 3 μm) analytical column (Thermo Scientific, Massachusetts, United States). The column oven temperature was set to 35°C while the autosampler temperature was adjusted to 4°C. The flow rate of the mobile phase was set at 0.25 ml/min and the injection volume was 20 µL. For all peptides, elution was achieved under a gradient program. The initial mobile phase condition consisted of water with 0.1% FA (solvent A) and ACN (solvent B) (95:5, v/v). Gradient elution steps were 5–50% B (0–12 min), 50–95% B (12–12.5 min), 95% B (12.5–13 min), 95–5% B (13–13.5 min) and 5% B (13.5–15 min).

The mass spectrometer was equipped with the electrospray ionization and operated in the positive ion mode to monitor the m/z transitions for all peptides and their internal standards. Mass spectrometry parameters such as declustering potential and collision energy were manually optimized for every single peptide and were summarized in Table 1. The following ion source parameters were applied: the ESI spray voltage was set at 4000 V; vaporizer temperature was set at 350°C; capillary temperature was set at 300°C; sheath gas pressure and aux gas pressure were set at 20 and 10, respectively.

### 2.5 Digestion procedure

The rat liver microsome was diluted in NH_4_HCO_3_ (50 mM, pH7.8) to a total protein concentration of 1 mg/ml 150 μL of diluted proteins were denatured at 95°C for 5 min and then added by 7.5 μL of DTT (500 mM) for the reduction of disulfide bonds in proteins. Samples were left at room temperature for 5 min and then incubated for 20 min at 60°C. Proteins were then alkylated with 15 μL of IAA (500 mM) and incubated at 37°C for 15 min in the dark. Samples were digested with trypsin at a trypsin/protein ratio of 1:40) at 37°C for 6 h. Digestion was terminated by adding 20 μL of ACN: Water: TFA (40:60:1, v/v/v). The mixture was vortexed for approximately 5 s and then centrifuged at 15,000 g for 10 min at 4°C. Internal standards solution was added to 160 μL of the clear supernatant and the mixed solution was evaporated to dryness under vacuum. The dried extract was re-suspended with 160 μL of ACN: Water: ACE: DMSO (15:80:5:0.5, v/v) and transferred to an injection vial for analysis. Liver microsome samples (IPhase Pharma Services) were used to optimize the digestion time in the above process, and six samples were measured in parallel at each time point. The standard curve and quality control samples were prepared by the above pretreatment steps after the standard solution was used to replace the rat liver microsomes.

### 2.6 Method validation

As shown in the current absolute quantitative analysis of proteomics, there is still no clear methodological guideline (Prasad et al., 2019). Therefore, we carried out the validation of the main content according to the bioanalytical guidelines combined with the needs of this research (FDA, 2018). We verified the accuracy, precision, linearity, stock solution stability, working solution stability, recovery, matrix effect and other parameters of the method.

### 2.7 Evaluation of matrix effect and recovery

The matrix effect was assessed by comparing the internal standard normalized response values of the standard solution group ( $R_{Standard}$ ) and the rat liver microsome-spiked group ( $R_{Spiked}$ ), and the latter needed to subtract the basal response ( $R_{Non-spiked}$ ) for comparison. The internal standard used for normalization was added during the final reconstitution in pretreatment. The specific calculation is shown in formula 1.

$$Matrix\;effect\;\left(\%\right)=\frac{R_{Spiked}/R_{Spiked\left(IS\right)}-R_{Non-spiked\;}/R_{Non-spiked\left(IS\right)}}{R_{Standard}/R_{Standard\left(IS\right)}}$$ (1)

The relative recovery was calculated by comparing the spiked group with the internal standard added before vacuum evaporation ( $R_{Spiked}^{\prime }$ ) and the spiked group with the internal standard added during reconstitution ( $R_{Spiked}$ ). Calculation was also performed using the base-subtracted response Eq. (2).

$$Recovery\;\left(\%\right)=\frac{R_{Spiked}^{\prime }/R_{Spiked\left(IS\right)}^{\prime }-R_{Non-Spiked}^{\prime }/R_{Non-spiked\left(IS\right)}^{\prime }}{R_{Spiked}/R_{Spiked\left(IS\right)}-R_{Non-spiked\;}/R_{Non-spiked\left(IS\right)}}$$ (2)

### 2.8 Enzyme activity detection by probe substrate method

The enzyme activity of CYPs was calculated according to the formation rate of the substrate metabolite. The substrate drugs and metabolism of seven CYP enzymes are phenacetin and acetaminophen (CYP1A2), bupropion and hydroxy-bupropion (CYP2B1), tolbutamide and 4-Hydroxy-tolbutamide (CYP2C6), phenytoin and 4-Hydroxymephenytoin (CYP2C11), dextromethorphan and dextrorphan (CYP2D1), chlorzoxazone and 6-Hydroxy-chlorzoxazone (CYP2E1), testosterone and 6β-Hydroxytestosterone (CYP3A1). LC-MS/MS detection method refers to previous research reports (He et al., 2007; Zhang et al., 2021). The probe drug and NADPH (1 mM) were mixed in 195 μL PBS (pH 7.4) for 1 min at 37°C. The enzymatic reaction was initiated by adding 5 µL of rat liver microsomes (protein 20 mg/ml), incubated for 30 min at 37°C, and then terminated by adding ice-cold methanol containing IS glibenclamide. The sample was centrifuged at 15,000 g for 10 min at 4°C and 5 μL supernatant was analyzed by LC-MS/MS to quantify the metabolites formed during these incubations. Each microsomal sample was assayed in triplicate.

### 2.9 Statistical analysis

The raw data was sorted using Microsoft Excel. Plotting used GraphPad Prism 8 (GraphPad Software Inc., San Diego, CA, United States). The Xcalibur software was used to establish the calibration curves fitted with weighted (1/X^2^) and to calculate the accuracy and precision of the QC samples for method validation (*n* = 6).

## 3 Results and discussion

### 3.1 Selection of surrogate peptide

Ideal surrogate peptides for metabolic enzymes require good chemical stability and specificity. Peptides of suitable length were first screened according to the trypsin cleavage site, and they do not contain labile amino acids such as cysteine, methionine, and tryptophan. Once selected, each peptide was evaluated with BLAST to confirm their specificity toward an isoform. According to parameters such as the E value, two surrogate peptides were finally selected for each metabolic enzyme, one for quantification and the other for qualitative research (Supplementary Table S1). For CYP3A1, although only one peptide was eligible for this study, it was fortunate that this peptide went well in the development and validation of the LC-MS/MS method. Furthermore, the surrogate peptide we used for CYP2E1 was consistent with those reported in a previous study (Ren et al., 2020), indicating the reproducibility of trypsin digestion across different laboratories. We also compared the surrogate peptides of the corresponding metabolic enzymes in rat and human liver microsomes, and found that the surrogate peptides of the reported enzyme isoforms were significantly different between species, except that the peptide of CYP2E1 was highly similar (Groer et al., 2014; Li and Zhu, 2020; Wenzel et al., 2021).

Stable isotope-labeled internal standards were synthesized for all surrogate peptides. With the ^13^C and ^15^N labeling, the molecular weight of the stable isotope-labeled peptide was increased by 7 Da for leucine (L), as well as isoleucine (I). The sequences of surrogate peptides containing deuterated amino acids were shown in Table 1.

### 3.2 LC-MS/MS optimization

The mass spectrometer was operated in positive mode using electrospray ionization. The standard solution was continuously injected into the liquid phase and mixed before entering the mass spectrometer, and then the mass spectrometry conditions were optimized. For maximum sensitivity, we used the m/z ratio of the precursor ion with the highest signal intensity as the Q1 filter setting. Among most of the surrogate peptides screened this time, the signal intensity of the triple-charged or double-charged precursor ions was dominant. The precursor ions were then scanned for product ions to identify fragments showing the highest signal intensities, and the following parameters were optimized to determine optimal fragmentation conditions: collision energy, declustering potential, entry potential and collision cell exit potential. Final acceptance of ions used for quantification also required blank testing to exclude possible matrix-induced interferences. The optimal parameters used in the analysis method are shown in Table 1. It is worth mentioning that for the surrogate peptide of CYP3A1, the amino-terminal glutamine undergoes the cyclization to pyroglutamate and loses 17 Da, thereby changing the triple-charged precursor ion from m/z 484.0 to 478.0 (actual value: 477.9) (Purwaha et al., 2014).

**FIGURE 1:**
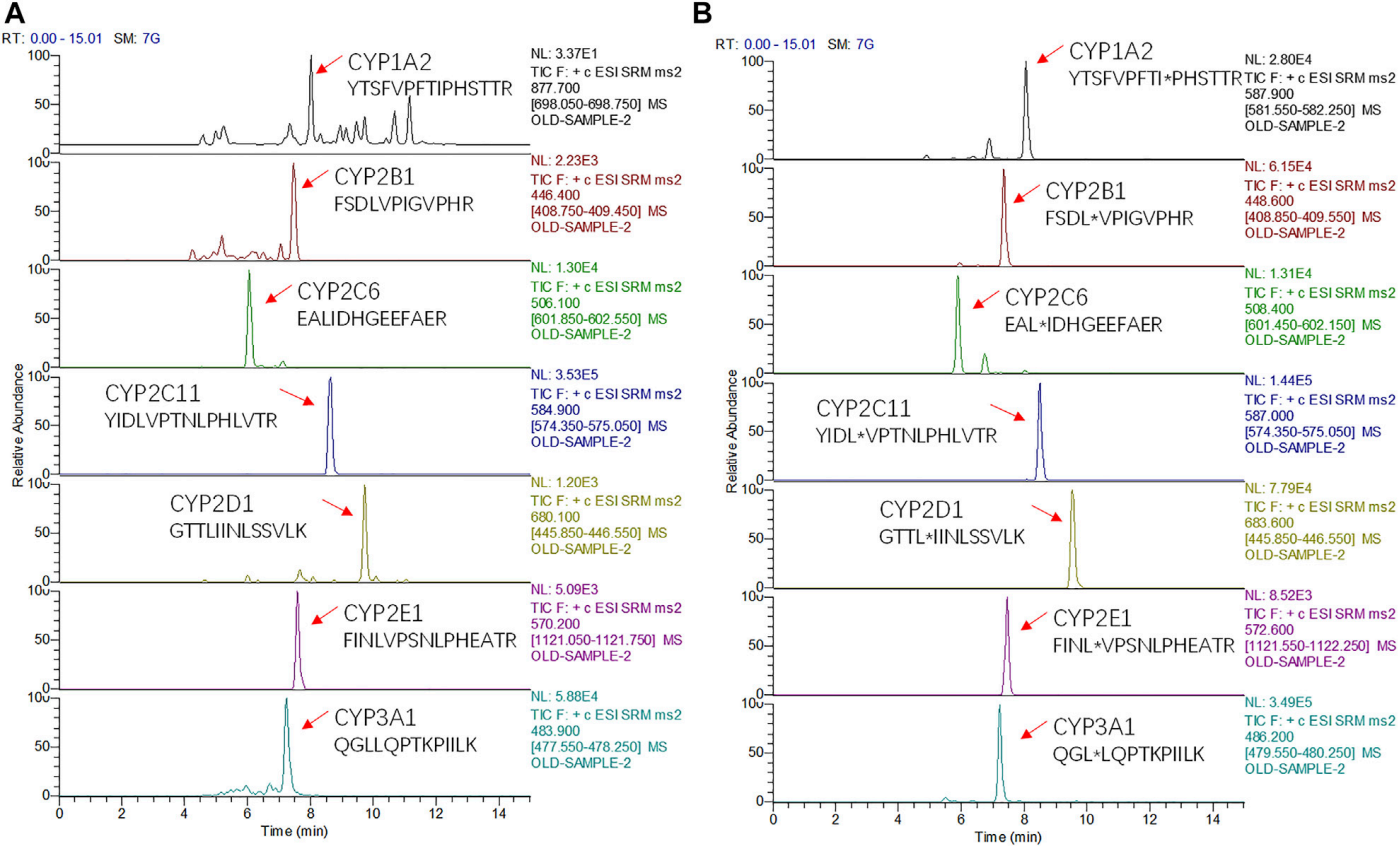
Chromatograms of seven surrogate peptides **(A)** and their corresponding isotopically-labeled peptides (*) **(B)** measured in mixed rat liver microsomes.

The principle of chromatographic optimization is to avoid the matrix effect on the premise of ensuring the chromatographic peak shape. To capture all seven peptides of interest in one analytical run and to consider the expected complexity of the digested samples, a 15 min gradient elution method was used and all surrogate peptides could be separated by chromatography (Figure 1, Supplementary Figure S1). The use of 0.1% formic acid as the aqueous phase avoided the poor peak shape associated with pure water and the reduced signal caused by volatile salts. Compared with methanol, using acetonitrile for the organic phase increased the elution power and reduced the column pressure. The method established in this study combined the advantages of analysis time and quantity, which was superior to other methods of the same type reported in other studies. For example, Yi Ren et al. reported that a 16.5-min gradient method was used to detect only CYP2E1 in rat liver microsomes (Ren et al., 2020). In studies of metabolic enzymes and/or transporters in human liver microsomes, these simultaneous assays took about an hour longer (Kawakami et al., 2011; Groer et al., 2014; Grangeon et al., 2019). Therefore, this study greatly improved the analysis efficiency.

### 3.3 Optimization of digestion

**FIGURE 2:**
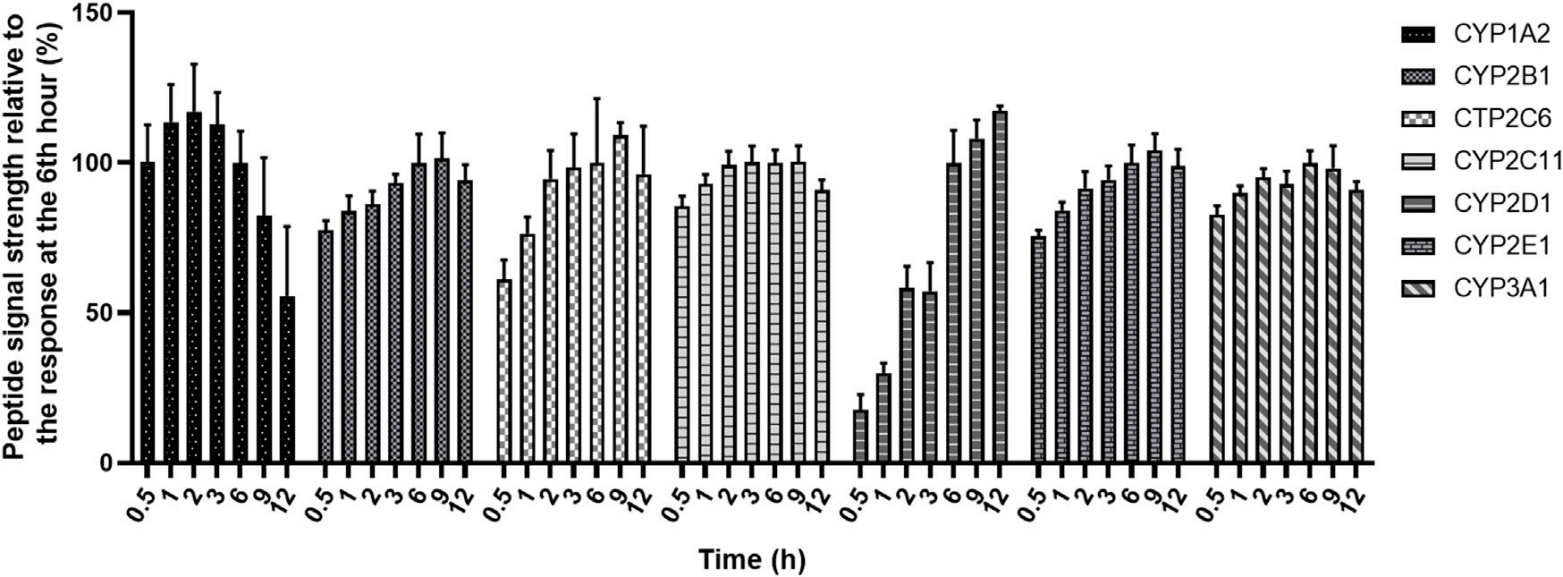
Peptide formation as a function of digestion time. Normalized calculations were performed with the response at the 6th hour as 100%. Six samples were measured in parallel at each time point.

The use of surrogate peptides as substitutes for quantifying target proteins requires robust and efficient digestion, which includes reduction, alkylation and digestion duration. The time required for trypsin digestion is crucial. Therefore, this parameter needed to be optimized, and the digestion time was set to 0.5, 1, 2, 3, 6, 9, and 12 h in this study. As shown in Figure 2, there were differences in the digestion profiles of seven CYP proteins. All the substituted peptides except CYP1A2 reached the content plateau at the 6th hour, and CYP2D1 content increased significantly with time until the 6th hour. However, the content of CYP1A2 has been relatively stable at 0.5 h, and has a downward trend from 6 h later. This phenomenon has also been reported by other studies (Grangeon et al., 2019; Ren et al., 2020). The stability experiment has proved that the peptide itself was stable during digestion (Table 3), so the possible reason for the decline may be the influence of its own digestive enzymes in rat liver microsomes. In conclusion, it was decided to terminate the digestion at the 6th hour.

**TABLE 3:** Stability of seven surrogate peptides. Results are expressed as percent nominal ±SD (*n* = 6).

Protein	Peptide	Conc	Stock solution (4 months, −80°C)	Standard working solution (1 week, -80°C)	Stability during digestion (6 h, 37°C)	In autosampler (12 h, 10°C)
		(nM)	%Nominal (mean ± SD)	%Nominal (mean ± SD)	%Nominal (mean ± SD)	%Nominal (mean ± SD)
CYP1A2	YTSFVPFTIPHSTTR	15	96.88 ± 9.41	99.21 ± 8.86	103.68 ± 5.04	104.85 ± 10.52
		150	98.85 ± 6.53	96.52 ± 8.61	102.47 ± 6.15	103.94 ± 6.22
		750	95.66 ± 4.03	102.23 ± 2.99	96.36 ± 7.96	99.73 ± 5.05
CYP2B1	FSDLVPIGVPHR	1.5	101.83 ± 8.97	98.70 ± 8.00	90.37 ± 7.02	105.46 ± 7.06
		15	101.53 ± 4.37	99.62 ± 5.81	92.09 ± 6.96	103.46 ± 4.74
		75	99.67 ± 5.93	99.97 ± 5.08	104.14 ± 4.20	103.75 ± 2.65
CYP2C6	EALIDHGEEFAER	6	108.03 ± 4.08	97.95 ± 7.49	102.72 ± 7.10	104.84 ± 12.60
		60	99.27 ± 8.92	99.45 ± 8.15	103.23 ± 7.32	112.76 ± 2.46
		300	103.38 ± 3.67	99.67 ± 6.83	102.80 ± 4.41	103.78 ± 8.46
CYP2C11	YIDLVPTNLPHLVTR	15	95.67 ± 2.18	107.26 ± 3.12	95.55 ± 5.26	111.18 ± 3.53
		150	102.42 ± 1.48	100.86 ± 5.45	90.85 ± 1.46	106.67 ± 4.02
		750	97.24 ± 2.69	97.27 ± 2.49	91.36 ± 4.42	102.22 ± 3.75
CYP2D1	GTTLIINLSSVLK	15	95.53 ± 4.37	100.29 ± 9.50	100.72 ± 8.13	100.32 ± 7.31
		150	96.77 ± 8.78	94.47 ± 4.87	102.16 ± 9.29	96.04 ± 5.43
		750	108.56 ± 5.33	111.13 ± 8.98	106.07 ± 1.62	112.42 ± 2.77
CYP2E1	FINLVPSNLPHEATR	6	105.74 ± 5.24	101.60 ± 9.15	96.63 ± 3.16	100.66 ± 9.82
		60	101.31 ± 8.05	107.21 ± 7.09	101.14 ± 7.30	99.02 ± 8.73
		300	94.76 ± 8.14	97.32 ± 5.14	89.64 ± 0.67	95.49 ± 5.93
CYP3A1	QGLLQPTKPIILK	1.5	95.47 ± 5.18	95.20 ± 5.21	99.91 ± 12.61	111.87 ± 3.36
		15	100.91 ± 3.68	103.24 ± 2.83	98.61 ± 3.60	107.38 ± 7.30
		75	100.37 ± 6.11	103.16 ± 6.18	95.81 ± 6.93	101.50 ± 2.84

### 3.4 Selection of blank matrix

The current LC-MS/MS-based CYP enzyme quantification methods mainly use the following three types of blank matrices to establish standard curves and QC samples: 1) Other tissues or serum of the same species or different species, such as 5% rat serum (Ren et al., 2020), human serum albumin (Groer et al., 2014), bovine serum albumin (Wenzel et al., 2021); 2) the same matrix, standard curve correction by subtracting the substrate (Sakamoto et al., 2011; Ohtsuki et al., 2012); 3) Standard solution without biological matrix (Grangeon et al., 2019). Serum protein is significantly different from that in liver microsomes, which may lead to different matrix effects affecting the quantitative analysis. If the standard curve is prepared using the same matrix or the same matrix diluted, the calculation process is complicated, and the subtraction of the blank matrix response may introduce bias, especially for low-concentration samples. Therefore, in this study, the influence of the matrix effect on quantification was significantly reduced by optimizing the chromatographic conditions, so that the standard curve prepared with the standard solution became simple and practical. Although the method still has a slight matrix effect and different degrees of absolute recovery (Supplementary Table S2), the isotope internal standard normalized matrix effect and relative recovery were all around 100% and the relative standard deviation was also less than 20% (Table 4). Therefore, in this method, it is reasonable and reliable to use a standard solution instead of liver microsomes to prepare standard curve and QC samples, and the internal standard for the above samples was added after the termination of digestion and before vacuum drying.

**TABLE 4:** Summary of internal standard normalized matrix effect and relative recovery in rat liver microsomes for all surrogate peptides. Results are expressed as percent nominal; RSD, relative standard deviation (*n* = 6).

Protein	Peptide	Conc	Matrix effect		Absolute recovery	
		(nM)	%Nominal	RSD (%)	%Nominal	RSD (%)
CYP1A2	YTSFVPFTIPHSTTR	15	111.14	12.5	98.55	14.1
		150	119.69	7.7	96.32	9.9
		750	117.96	5.8	98.89	11.6
CYP2B1	FSDLVPIGVPHR	1.5	111.48	16.9	95.59	16.8
		15	98.40	5.0	91.94	5.1
		75	99.41	5.0	95.14	7.1
CYP2C6	EALIDHGEEFAER	6	119.36	15.2	103.19	3.9
		60	98.33	7.8	90.64	8.0
		300	100.92	12.2	91.77	6.4
CYP2C11	YIDLVPTNLPHLVTR	15	93.59	17.2	107.23	6.1
		150	81.05	9.1	104.87	11.6
		750	95.31	9.4	94.24	11.2
CYP2D1	GTTLIINLSSVLK	15	107.25	8.5	111.40	14.4
		150	118.57	11.7	109.98	10.9
		750	118.40	8.6	109.30	12.3
CYP2E1	FINLVPSNLPHEATR	6	108.97	14.0	101.79	18.6
		60	98.61	14.6	91.68	13.7
		300	96.21	12.0	98.79	6.2
CYP3A1	QGLLQPTKPIILK	1.5	98.42	8.6	107.05	15.8
		15	94.22	3.4	97.53	10.4
		75	95.51	4.3	98.02	5.9

### 3.5 Method validation

#### 3.5.1 LLOQ and linearity

The method was found to be linear over the appropriate calibration range for all surrogate peptides. Linear regression (weighted 1/X^2^) yielded the best fit of the concentration-response relationship. During assay validation, the correlation coefficient (r^2^) for all calibration curves ranged between 0.985 and 1.0 (Supplementary Table S3). Therefore, the LLOQ was set 5 nM (CYP1A2, 2C11 and 2D1), 0.5 nM (CYP2B1 and 3A1), or 2 nM (CYP2C6 and 2E1) (Table 2). For LLOQ, the precision was better than 15%, and the accuracy was between 80.0–120%.

#### 3.5.2 Precision and accuracy

The precision and accuracy were assessed by observing the analysis of QC samples at three concentrations (low, medium and high) in three analytical runs (*n* = 6). Precision was reflected by the relative standard deviation (RSD) and should be better than 15%. Accuracy was assessed using relative error (RE) and should be within the recommended range of acceptance (85.0–115.0%). Intra- and inter-batch precision and accuracy for all surrogate peptides met the above requirements. The results were shown in Table 2.

#### 3.5.3 Stability

To assess the stability of the surrogate peptides, we performed several stability experiments. For stability data, the precision (*n* = 6) should not exceed 15% and the mean accuracy value should be within ±15% of the nominal value. Stock stability was assessed by comparing stocks stored at −80°C for four months with fresh stocks. QC samples were stored at −80°C for 1 week to assess the short-term stability of the peptides. After trypsin digestion for six hours, the QC concentration peptides were compared with the QC samples without this process to investigate the stability of these peptides in the digestion process. The QC samples were placed in the autosampler for 12 h and compared with the freshly prepared samples for the stability test in the autosampler. All stability results met the above criteria and indicated that all peptides were stable during the method validation (Table 3).

### 3.6 Determination of CYPs in rat liver microsomes

This absolute quantification method was successfully applied to the quantification of seven CYP isoforms in commercial mixed rat liver microsomes from four different sources (Table 5). The abundance of the metabolic enzymes including CYP2B1, 2C11, 2D1 and 3A1 in rat liver microsomes from these four sources was significantly different. The results of CYPs determined from sources 1 and 2 were similar, and CYP2C11 was the highest. The content of CYP2C11 in source 3 was low, while the result in source 4 was lower than the lower limit of quantification (6.25 pmol/mg). Since CYP2C11 is not expressed in immature rats and is induced dramatically at puberty (beginning 4–5 weeks of age) in male rats (Martignoni et al., 2006), we speculated that the age of rats was the main factor leading to the low content of CYP2C11 in 3 and 4 sources. For the content of CYPs in liver microsomes of SD rats, CYP2E1 has been reported to be about 5–25 pmol/mg, which is higher than the enzyme content in liver S9 fractions (2–8 pmol/mg) (Ren et al., 2020). The CYP2E1 content determined in this study was close to the upper limit of the content reported above. In the study of Hammer H et al., the contents of CYP1A2, 2B1/2, 2C11 and 2E1 in liver tissue samples of male Wistar rats were 1.3, 0.2, 58 and 5.9 fmol/μg (pmol/mg) respectively (Hammer et al., 2021). These results were lower than those of this study. In addition to species, sample types should be a key factor in the difference between the two studies.

**TABLE 5:** Protein amounts of CYP enzymes as observed in pooled rat liver microsomes from four sources (IPhase Pharma Services, Corning Gentest, PrimeTox and Meilunbio). Results are expressed as concentration (Mean ± SD) (*n* = 4).

Protein	Peptide	Protein amount (pmol/mg of protein, mean ± SD)			
		Source 1	Source 2	Source 3	Source 4
CYP1A2	YTSFVPFTIPHSTTR	-	-	-	-
CYP2B1	FSDLVPIGVPHR	1.02 ± 0.05	2.06 ± 0.05	39.77 ± 1.52	-
CYP2C6	EALIDHGEEFAER	60.25 ± 4.46	82.11 ± 2.72	80.65 ± 3.95	71.34 ± 7.31
CYP2C11	YIDLVPTNLPHLVTR	239.46 ± 10.89	258.00 ± 8.73	61.34 ± 2.55	-
CYP2D1	GTTLIINLSSVLK	173.52 ± 10.35	58.36 ± 5.79	99.76 ± 9.63	77.29 ± 10.01
CYP2E1	FINLVPSNLPHEATR	26.94 ± 0.51	34.68 ± 1.23	33.85 ± 1.78	27.52 ± 1.20
CYP3A1	QGLLQPTKPIILK	18.69 ± 0.99	14.77 ± 0.76	35.86 ± 0.75	31.06 ± 2.24

**FIGURE 3:**
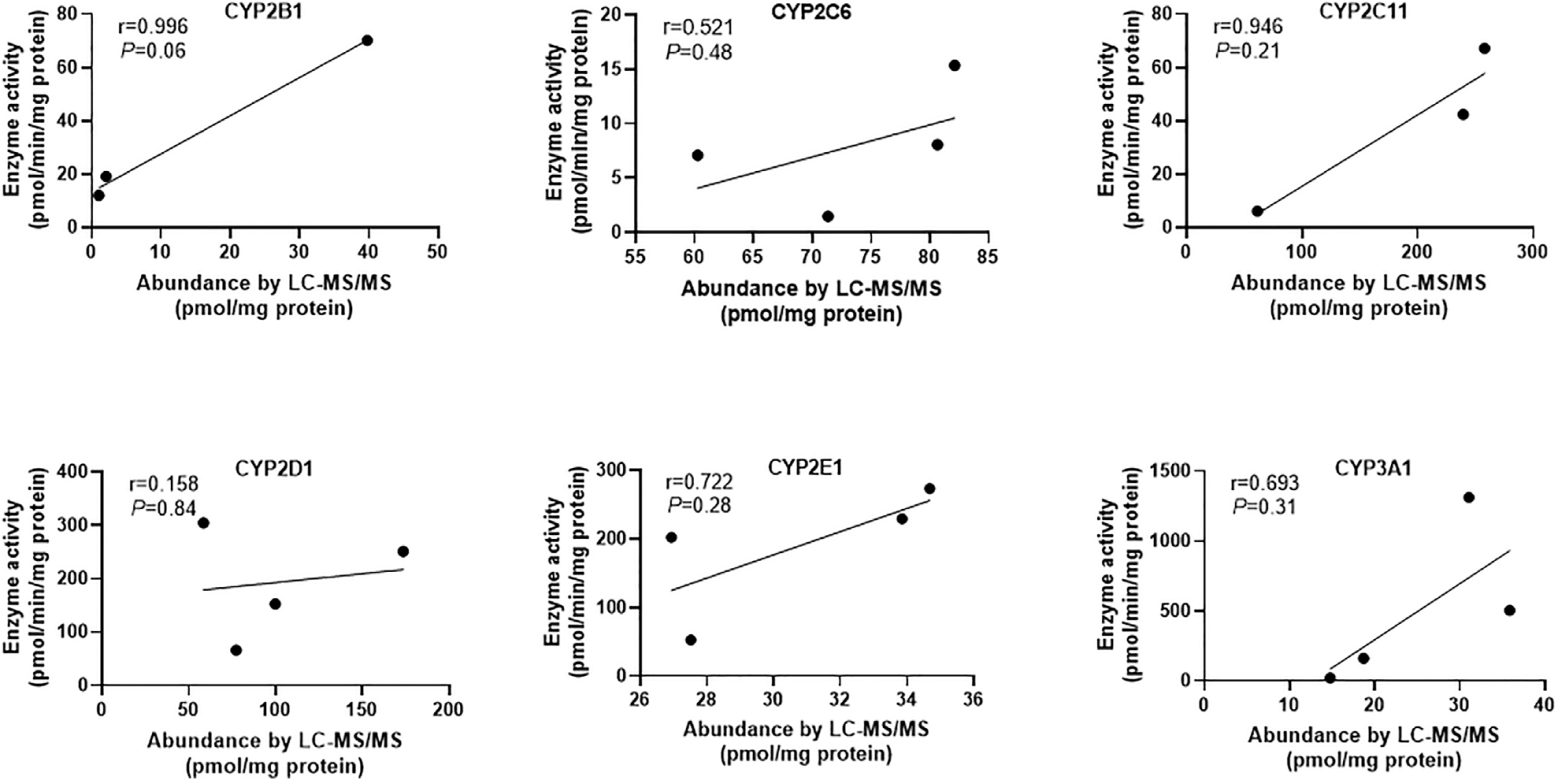
The correlation analysis between protein abundance by LC-MS/MS and enzyme activity of six CYP enzymes. Since the protein amounts of CYP1A2 from different sources were lower than the lower limit of quantification, the correlation analysis between its content and activity has not been carried out. Pearson correlation analysis was performed and *p* < 0.05 was considered statistically significant.

In addition, the relationship between the abundance and activity of CYPs was also studied. The results showed that the content and activity of each CYP enzyme had a positive correlation trend, but there was no statistical significance (Figure 3). The reasons for the above phenomena include: 1) the absolute protein quantitative method based on LC-MS/MS has very high specificity for CYP isoforms, while the drug indicating enzyme activity in the probe substrate method usually lacks sufficient specificity, so there is a potential deviation in the results of enzyme activity (Giri et al., 2019); 2) The number of samples measured in this study was small, so it is necessary to expand the samples to further explore the law. It has been reported that immunological methods including WB are not specific enough for the determination of CYPs content, which may lead to contradictory conclusions (Ren et al., 2020). LC-MS/MS method has the advantages of high specificity and high throughput. Therefore, the method established in this study will greatly improve the reliability of related research in the field of drug metabolism based on the rat liver microsomal model.

## 4 Conclusion

In the present work, we developed and validated an LC-MS/MS-based targeted proteomics for simultaneous absolute quantification of seven major CYP enzymes in rat liver microsomes. The optimized pretreatment and chromatographic conditions ensured the robustness of the method, and the assays were validated for selectivity, linearity, matrix effect, recovery, stability, precision and accuracy. Finally, this method was successfully applied to the detection of rat liver microsome samples from four sources. This study provides a high-throughput and stable technical basis for the study of drug metabolism in the rat.

## Data Availability

The original contributions presented in the study are included in the article/Supplementary Material, further inquiries can be directed to the corresponding authors.

## Abbreviations

ACE, acetic acid; CYP, cytochrome P450; DMSO, dimethyl sulfoxide; DTT, dithiothreitol; ELISA, enzyme linked immunosorbent assay; FA, formic acid; IAA, iodoacetamide; LC-MS/MS, liquid chromatography-tandem mass spectrometry; RT-PCR, real-time polymerase chain reaction; TFA, trifluoroacetic acid; WB, Western blotting.

## References

[B1] An B. Zhang M. Pu J. Shen S. Qu Y. Chen Y. J. (2019). High-throughput, sensitive LC-MS quantification of biotherapeutics and biomarkers using antibody-free, peptide-level, multiple-mechanism enrichment via strategic regulation of pH and ionic and solvent strengths. Anal. Chem. 91 (5), 3475–3483. 10.1021/acs.analchem.8b05046 30712341

[B2] Bhatt D. K. Prasad B. (2018). Critical issues and optimized practices in quantification of protein abundance level to determine interindividual variability in DMET proteins by LC-MS/MS proteomics. Clin. Pharmacol. Ther. 103 (4), 619–630. 10.1002/cpt.819 28833066 PMC5816727

[B3] Couto N. Al-Majdoub Z. M. Gibson S. Davies P. J. Achour B. Harwood M. D. (2020). Quantitative proteomics of clinically relevant drug-metabolizing enzymes and drug transporters and their intercorrelations in the human small intestine. Drug Metab. Dispos. 48 (4), 245–254. 10.1124/dmd.119.089656 31959703 PMC7076527

[B4] De Nicolo A. Cantu M. D'Avolio A. (2017). Matrix effect management in liquid chromatography mass spectrometry: the internal standard normalized matrix effect. Bioanalysis 9 (14), 1093–1105. 10.4155/bio-2017-0059 28737421

[B5] Dostalek M. Court M. H. Yan B. F. Akhlaghi F. (2011). Significantly reduced cytochrome P450 3A4 expression and activity in liver from humans with diabetes mellitus. Br. J. Pharmacol. 163 (5), 937–947. 10.1111/j.1476-5381.2011.01270.x 21323901 PMC3130941

[B6] Fda (2018). Bioanalytical method validation guidance for industry. Available at: https://www.fda.gov/regulatory-information/search-fda-guidance-documents/bioanalytical-method-validation-guidance-industry .

[B7] Giri P. Patel H. Srinivas N. R. (2019). Use of cocktail probe drugs for indexing cytochrome P450 enzymes in clinical Pharmacology studies - review of case studies. Drug Metab. Lett. 13 (1), 3–18. 10.2174/1872312812666181119154734 30451124

[B8] Grangeon A. Clermont V. Barama A. Gaudette F. Turgeon J. Michaud V. (2019). Development and validation of an absolute protein assay for the simultaneous quantification of fourteen CYP450s in human microsomes by HPLC-MS/MS-based targeted proteomics. J. Pharm. Biomed. Anal. 173, 96–107. 10.1016/j.jpba.2019.05.006 31125949

[B9] Groer C. Busch D. Patrzyk M. Beyer K. Busemann A. Heidecke C. D. (2014). Absolute protein quantification of clinically relevant cytochrome P450 enzymes and UDP-glucuronosyltransferases by mass spectrometry-based targeted proteomics. J. Pharm. Biomed. Anal. 100, 393–401. 10.1016/j.jpba.2014.08.016 25218440

[B10] Hammer H. Schmidt F. Marx-Stoelting P. Potz O. Braeuning A. (2021). Cross-species analysis of hepatic cytochrome P450 and transport protein expression. Arch. Toxicol. 95 (1), 117–133. 10.1007/s00204-020-02939-4 33150952 PMC7811513

[B11] He F. Bi H. C. Xie Z. Y. Zuo Z. Li J. K. Li X. (2007). Rapid determination of six metabolites from multiple cytochrome P450 probe substrates in human liver microsome by liquid chromatography/mass spectrometry: application to high-throughput inhibition screening of terpenoids. Rapid Commun. Mass Spectrom. 21 (5), 635–643. 10.1002/rcm.2881 17279482

[B12] Kawakami H. Ohtsuki S. Kamiie J. Suzuki T. Abe T. Terasaki T. (2011). Simultaneous absolute quantification of 11 cytochrome P450 isoforms in human liver microsomes by liquid chromatography tandem mass spectrometry with *in silico* target peptide selection. J. Pharm. Sci. 100 (1), 341–352. 10.1002/jps.22255 20564338

[B13] Li J. Zhu H. J. (2020). Liquid chromatography-tandem mass spectrometry (LC-MS/MS)-Based proteomics of drug-metabolizing enzymes and transporters. Molecules 25 (11), E2718. 10.3390/molecules25112718 32545386 PMC7321193

[B14] Li Q. Jiang F. Guan Y. Jiang X. Wu J. Huang M. (2022). Development, validation and application of a UHPLC-MS/MS method for quantification of the adiponectin-derived active peptide ADP355 in rat plasma. Biomed. Chromatogr. 36, e5358. 10.1002/bmc.5358 35187696

[B15] Liu Q. Jiang F. Zhu J. Zhong G. Huang M. (2019). Development, validation, and application of a New method to correct the nonlinearity problem in LC-MS/MS quantification using stable isotope-labeled internal standards. Anal. Chem. 91 (15), 9616–9622. 10.1021/acs.analchem.9b00947 31268297

[B16] Manikandan P. Nagini S. (2018). Cytochrome P450 structure, function and clinical significance: a review. Curr. Drug Targets 19 (1), 38–54. 10.2174/1389450118666170125144557 28124606

[B17] Martignoni M. Groothuis G. M. M. de Kanter R. (2006). Species differences between mouse, rat, dog, monkey and human CYP-mediated drug metabolism, inhibition and induction. Expert Opin. Drug Metab. Toxicol. 2 (6), 875–894. 10.1517/17425255.2.6.875 17125407

[B18] Ohtsuki S. Schaefer O. Kawakami H. Inoue T. Liehner S. Saito A. (2012). Simultaneous absolute protein quantification of transporters, cytochromes P450, and UDP-glucuronosyltransferases as a novel approach for the characterization of individual human liver: comparison with mRNA levels and activities. Drug Metab. Dispos. 40 (1), 83–92. 10.1124/dmd.111.042259 21994437

[B19] Pearce R. E. Gaedigk R. Twist G. P. Dai H. Riffel A. K. Leeder J. S. (2016). Developmental expression of CYP2B6: A comprehensive analysis of mRNA expression, protein content and bupropion hydroxylase activity and the impact of genetic variation. Drug Metab. Dispos. 44 (7), 948–958. 10.1124/dmd.115.067546 26608082 PMC4931886

[B20] Prasad B. Achour B. Artursson P. Hop C. Lai Y. Smith P. C. (2019). Toward a consensus on applying quantitative liquid chromatography-tandem mass spectrometry proteomics in translational Pharmacology research: a white paper. Clin. Pharmacol. Ther. 106 (3), 525–543. 10.1002/cpt.1537 31175671 PMC6692196

[B21] Purwaha P. Silva L. P. Hawke D. H. Weinstein J. N. Lorenzi P. L. (2014). An artifact in LC-MS/MS measurement of glutamine and glutamic acid: in-source cyclization to pyroglutamic acid. Anal. Chem. 86 (12), 5633–5637. 10.1021/ac501451v 24892977 PMC4063328

[B22] Ren Y. Ding Y. Meng F. Jiang L. Li H. Huang J. (2020). Quantification of CYP2E1 in rat liver by UPLC-MS/MS-based targeted proteomics assay: a novel approach for enzyme activity assessment. Anal. Bioanal. Chem. 412 (22), 5409–5418. 10.1007/s00216-020-02757-8 32588109

[B23] Sakamoto A. Matsumaru T. Ishiguro N. Schaefer O. Ohtsuki S. Inoue T. (2011). Reliability and robustness of simultaneous absolute quantification of drug transporters, cytochrome P450 enzymes, and Udp-glucuronosyltransferases in human liver tissue by multiplexed MRM/selected reaction monitoring mode tandem mass spectrometry with nano-liquid chromatography. J. Pharm. Sci. 100 (9), 4037–4043. 10.1002/jps.22591 21544820

[B24] Shao Y. Yin X. Kang D. Shen B. Zhu Z. Li X. (2017). An integrated strategy for the quantitative analysis of endogenous proteins: a case of gender-dependent expression of P450 enzymes in rat liver microsome. Talanta 170, 514–522. 10.1016/j.talanta.2017.04.050 28501205

[B25] Singh D. Kashyap A. Pandey R. V. Saini K. S. (2011). Novel advances in cytochrome P450 research. Drug Discov. Today 16 (17-18), 793–799. 10.1016/j.drudis.2011.08.003 21864709

[B26] Tan A. Levesque I. A. Levesque I. M. Viel F. Boudreau N. Levesque A. (2011). Analyte and internal standard cross signal contributions and their impact on quantitation in LC-MS based bioanalysis. J. Chromatogr. B Anal. Technol. Biomed. Life Sci. 879 (21), 1954–1960. 10.1016/j.jchromb.2011.05.027 21680265

[B27] van Midwoud P. M. Rieux L. Bischoff R. Verpoorte E. Niederlander H. A. (2007). Improvement of recovery and repeatability in liquid chromatography-mass spectrometry analysis of peptides. J. Proteome Res. 6 (2), 781–791. 10.1021/pr0604099 17269734

[B28] Wegler C. Gaugaz F. Z. Andersson T. B. Wisniewski J. R. Busch D. Groer C. (2017). Variability in mass spectrometry-based quantification of clinically relevant drug transporters and drug metabolizing enzymes. Mol. Pharm. 14 (9), 3142–3151. 10.1021/acs.molpharmaceut.7b00364 28767254

[B29] Wenzel C. Drozdzik M. Oswald S. (2021). Mass spectrometry-based targeted proteomics method for the quantification of clinically relevant drug metabolizing enzymes in human specimens. J. Chromatogr. B Anal. Technol. Biomed. Life Sci. 1180, 122891. 10.1016/j.jchromb.2021.122891 34390906

[B30] Xie F. Ding X. Zhang Q. Y. (2016). An update on the role of intestinal cytochrome P450 enzymes in drug disposition. Acta Pharm. Sin. B 6 (5), 374–383. 10.1016/j.apsb.2016.07.012 27709006 PMC5045550

[B31] Zanger U. M. Schwab M. (2013). Cytochrome P450 enzymes in drug metabolism: regulation of gene expression, enzyme activities, and impact of genetic variation. Pharmacol. Ther. 138 (1), 103–141. 10.1016/j.pharmthera.2012.12.007 23333322

[B32] Zhang H. F. Wang H. H. Gao N. Wei J. Y. Tian X. Zhao Y. (2016). Physiological content and intrinsic activities of 10 cytochrome P450 isoforms in human normal liver microsomes. J. Pharmacol. Exp. Ther. 358 (1), 83–93. 10.1124/jpet.116.233635 27189963

[B33] Zhang Y. J. Zhou W. L. Yu F. Wang Q. Peng C. Kan J. Y. (2021). Evaluation of the effect of bovis calculus artifactus on eight rat liver cytochrome P450 isozymes using LC-MS/MS and cocktail approach. Xenobiotica. 51 (9), 1010–1018. 10.1080/00498254.2021.1959673 34294011

