# Supplementary material for: Simultaneous absolute protein quantification of seven cytochrome P450 isoforms in rat liver microsomes by LC-MS/MS-based isotope internal standard method
Source: Front Pharmacol. 2022 Aug 17;13:906027. doi: 10.3389/fphar.2022.906027 (PMC9428253; doi:10.3389/fphar.2022.906027)
Supplement: Supplementary file 1 [file DataSheet1.DOCX]

**Simultaneous Absolute Protein Quantification of Seven Cytochrome P450 Isoforms in Rat Liver Microsomes by LC-MS/MS-based Isotope Internal Standard Method**

Fulin Jiang^1#^, Chang Zhang^1#^, Zihan Lu^1^, Jingyu Liu^1^, Peiqing Liu^2^, Min Huang^1^*, Guoping Zhong^1^*

1. Institute of Clinical Pharmacology, Guangdong Provincial Key Laboratory of New Drug Design and Evaluation, School of Pharmaceutical Sciences, Sun Yat-sen University, Guangzhou, China.

2. School of Pharmaceutical Sciences, National and Local United Engineering Lab of Druggability and New Drugs Evaluation, Sun Yat-sen University, Guangzhou, China.

^#^These first authors contributed equally to this work and share first authorship.

*These corresponding authors contributed equally.

**Contents**

In this supporting document more detailed information about the parameters of the specificity (Table S1), matrix effect and absolute recovery (Table S2), standard curves (Table S3) and chromatogram of blank sample and lower limit of quantitation (Figure S1). This information is ordered by order of mention in the text.

TABLE S1. The parameters of the specificity of the peptide by BLAST.

| Protein | Peptide | Max Score | Total Score | Query Cover | E value |
| --- | --- | --- | --- | --- | --- |
| CYP1A2 | YTSFVPFTIPHSTTR | 52.4 | 52.4 | 1 | 0.000002 |
|  | NFNDNFVLFLQK | 43.5 | 43.5 | 1 | 0.002 |
| CYP2B1 | FSDLVPIGVPHR | 41.8 | 41.8 | 1 | 0.007 |
|  | EALVGQAEDFSGR | 42.6 | 42.6 | 1 | 0.004 |
| CYP2C6 | EALIDHGEEFAER | 45.2 | 45.2 | 1 | 0.0005 |
|  | EHQESLDVTNPR | 42.2 | 42.2 | 1 | 0.005 |
| CYPC11 | YIDLVPTNLPHLVTR | 52.4 | 52.4 | 1 | 0.000002 |
|  | EALVDLGEEFSGR | 43.1 | 43.1 | 1 | 0.003 |
| CYP2D1 | GTTLIINLSSVLK | 41.8 | 41.8 | 1 | 0.008 |
|  | NLTDAFLAEVEK | 40.5 | 40.5 | 1 | 0.019 |
| CYP2E1 | FINLVPSNLPHEATR | 51.5 | 51.5 | 1 | 0.000004 |
|  | FKPEHFLNENGK | 43.1 | 43.1 | 1 | 0.002 |
| CYP3A1 | QGLLQPTKPIILK | 43.9 | 43.9 | 1 | 0.001 |

TABLE S2. Summary of matrix effect and absolute recovery in rat liver microsomes for all surrogate peptides. Results are expressed as percent nominal; RSD, relative standard deviation (n = 6).

| Protein | Peptide |  | Conc. | Matrix effect | |  | Absolute recovery | |
| --- | --- | --- | --- | --- | --- | --- | --- | --- |
|  |  |  | (nM) | %Nominal | RSD (%) |  | %Nominal | RSD (%) |
| CYP1A2 | YTSFVPFTIPHSTTR |  | 15 | 139.23 | 11.1 |  | 75.84 | 12.4 |
|  |  |  | 150 | 151.04 | 5.6 |  | 109.05 | 13.9 |
|  |  |  | 750 | 126.84 | 5.5 |  | 101.59 | 10.8 |
| CYP2B1 | FSDLVPIGVPHR |  | 1.5 | 123.31 | 18.0 |  | 99.98 | 19.9 |
|  |  |  | 15 | 108.00 | 3.2 |  | 101.20 | 9.4 |
|  |  |  | 75 | 106.00 | 3.2 |  | 96.5 | 13.9 |
| CYP2C6 | EALIDHGEEFAER |  | 6 | 123.52 | 17.5 |  | 102.28 | 13.4 |
|  |  |  | 60 | 103.32 | 4.1 |  | 101.80 | 8.2 |
|  |  |  | 300 | 98.30 | 2.4 |  | 96.33 | 11.0 |
| CYP2C11 | YIDLVPTNLPHLVTR |  | 15 | 106.14 | 22.7 |  | 136.36 | 24.3 |
|  |  |  | 150 | 109.97 | 32.8 |  | 116.10 | 11.5 |
|  |  |  | 750 | 135.75 | 11.0 |  | 85.8 | 23.6 |
| CYP2D1 | GTTLIINLSSVLK |  | 15 | 76.00 | 15.1 |  | 90.50 | 27.7 |
|  |  |  | 150 | 91.35 | 10.4 |  | 106.80 | 10.5 |
|  |  |  | 750 | 126.90 | 4.4 |  | 86.31 | 30.3 |
| CYP2E1 | FINLVPSNLPHEATR |  | 6 | 137.17 | 19.2 |  | 123.78 | 10.6 |
|  |  |  | 60 | 109.65 | 11.4 |  | 103.85 | 11.6 |
|  |  |  | 300 | 106.07 | 2.4 |  | 97.73 | 14.8 |
| CYP3A1 | QGLLQPTKPIILK |  | 1.5 | 136.98 | 24.9 |  | 80.09 | 21.6 |
|  |  |  | 15 | 110.90 | 6.3 |  | 105.32 | 15.3 |
|  |  |  | 75 | 107.34 | 3.3 |  | 98.99 | 15.8 |

TABLE S3. Standard curve of seven surrogate peptides.

| Protein | Peptide | linear | r^2^ |
| --- | --- | --- | --- |
| CYP1A2 | YTSFVPFTIPHSTTR | Y = -0.00272471+0.00989219*X | 0.9978 |
| CYP2B1 | FSDLVPIGVPHR | Y = -0.00385403+0.0162608*X | 0.9935 |
| CYP2C6 | EALIDHGEEFAER | Y = -0.00157544+0.00768691*X | 0.9906 |
| CYP2C11 | YIDLVPTNLPHLVTR | Y = 0.00419+0.016945*X | 0.9926 |
| CYP2D1 | GTTLIINLSSVLK | Y = 0.0366403+0.0124409*X | 0.9899 |
| CYP2E1 | FINLVPSNLPHEATR | Y = -6.48204e-005+0.0239933*X | 0.9964 |
| CYP3A1 | QGLLQPTKPIILK | Y = 9.56689e-005+0.00358485*X | 0.9900 |

FIGURE S1. Chromatogram of blank sample (A) and lower limit of quantitation (B).
